# Heterologous DNA Uptake in Cultured *Symbiodinium* spp. Aided by *Agrobacterium tumefaciens*

**DOI:** 10.1371/journal.pone.0132693

**Published:** 2015-07-13

**Authors:** Mario Fernando Ortiz-Matamoros, Tania Islas-Flores, Boris Voigt, Diedrik Menzel, František Baluška, Marco A. Villanueva

**Affiliations:** 1 Unidad Académica de Sistemas Arrecifales, Instituto de Ciencias del Mar y Limnología, Universidad Nacional Autónoma de México, Prol. Avenida Niños Héroes S/N, Puerto Morelos, Quintana Roo, 77580, México; 2 Posgrado en Ciencias del Mar y Limnología, Instituto de Ciencias del Mar y Limnología, Universidad Nacional Autónoma de México, Circuito Exterior S/N, Ciudad Universitaria, Delegación Coyoacán, Distrito Federal, 04510, México; 3 Institut für Zellulär und Molekulare Botanik Universität Bonn, Kirschallee 1, Bonn, D-53115, Germany; King Abdullah University of Science and Technology, SAUDI ARABIA

## Abstract

Plant-targeted pCB302 plasmids containing sequences encoding *gfp* fusions with a microtubule-binding domain; *gfp* with the fimbrin actin-binding domain 2; and *gfp* with *AtRACK1C* from *Arabidopsis thaliana*, all harbored in *Agrobacterium tumefaciens*, were used to assay heterologous expression on three different clades of the photosynthetic dinoflagellate, *Symbiodinium*. Accessibility to the resistant cell wall and through the plasma membrane of these dinoflagellates was gained after brief but vigorous shaking in the presence of glass beads and polyethylene glycol. A resistance gene to the herbicide Basta allowed appropriate selection of the cells expressing the hybrid proteins, which showed a characteristic green fluorescence, although they appeared to lose their photosynthetic pigments and did not further divide. Cell GFP expression frequency measured as green fluorescence emission yielded 839 per every 10^6^ cells for *Symbiodinium kawagutii*, followed by 640 and 460 per every 10^6^ cells for *Symbiodinium microadriaticum* and *Symbiodinium* sp. Mf11, respectively. Genomic PCR with specific primers amplified the *AtRACK1C* and *gfp* sequences after selection in all clades, thus revealing their presence in the cells. RT-PCR from RNA of *S*. *kawagutii* co-incubated with *A*. *tumefaciens* harboring each of the three vectors with their respective constructs, amplified products corresponding to the heterologous *gfp* sequence while no products were obtained from three distinct negative controls. The reported procedure shows that mild abrasion followed by co-incubation with *A*. *tumefaciens* harboring heterologous plasmids with *CaMV35S* and *nos* promoters can lead to expression of the encoded proteins into the *Symbiodinium* cells in culture. Despite the obvious drawbacks of the procedure, this is an important first step towards a stable transformation of *Symbiodinium*.

## Introduction

Photosynthetic dinoflagellates of the genus *Symbiodinium* live in symbiosis within a wide phyletic range of marine invertebrate hosts including cnidarians, mollusks, porifera and platyhelminthes. Photosynthetic *Symbiodinium* are obligate symbionts of reef-building corals and thus, coral reefs are highly dependent on the photosynthetic machinery of these microorganisms [1]. Many studies that attempt to dissect the multiple pathways by which physiological and symbiotic interaction mechanisms are controlled and regulated in these photosynthetic microorganisms have been carried out. For example, there have been reports on the effect of elevated temperature in symbiosis [2], ROS-mediated bleaching [3, 4], origin of the symbiosome [5], coral-dinoflagellate recognition [6], and genetic differences in *Symbiodinium* [7], among others. However, due to the lack of appropriate tools for functional genomics applications, all these studies have been, for the most part, carried out using biochemical and cell biological techniques. In fact, molecular biology techniques have only been applied recently and to a limited extent [8–15], but no significant functional genomics research has been carried out. This is greatly due to the fact that only one report of stable transformation of the dinoflagellates *Symbiodinium* and *Amphidinium* in culture exists since 1998 [16]; and more recently, our own effort reporting the transient transformation of three *Symbiodinium* clades [17].

Transformation techniques provide a powerful tool that allows the introduction and expression of foreign genes into a living organism for such functional genomics studies. Thus, reproducible and reliable genetic transformation methods are a key tool for understanding the physiology and cell biology of these photosynthetic dinoflagellates. More importantly, these tools are essential to dissect and understand the cellular and molecular mechanisms underlying the cnidarian–dinoflagellate symbiosis such as inter-partner signaling, coordination of cell division, control of nutrient transport, and to identify target genes and their associated pathways involved in the establishment and maintenance of symbiosis. The successful transformation of many organisms including microalgae has been reported in recent years [18–20]. In addition, the standard stable transformation of plants is mediated by *Agrobacterium tumefaciens*, a gram-negative, plant-pathogenic soil bacterium, which has been widely used to introduce DNA in the plant genome [21–25]. Interestingly, the host range of *Agrobacterium* has been extended more recently to non-plant eukaryotic organisms under laboratory conditions. These include yeast [26], green algae [27], filamentous fungi [28], cultivated mushrooms [29], and even human cultured cells [30]. This approach has not been applied to attempt to transform *Symbiodinium* cells to date, even though it is imperative to develop and apply the appropriate genetic engineering techniques that will allow major advances in understanding the basic biology of this organism. Moreover, transformation tools are a must now that the first *Symbiodinium* genome draft has been released [31]. Here, we report a simple method of *A*. *tumefaciens*-aided entry and expression of foreign genes encoding three different GFP-fused proteins in three different *Symbiodinium* clades. Expression of the foreign genes using this procedure led to apparent loss of the photosynthetic pigments and a quiescent-like stage of the cells, imposing a limitation of the protocol. Nevertheless, this is the first report of the use of *A*. *tumefaciens* to assist heterologous gene expression in these dinoflagellates, and despite the observed limitations, it represents an important step towards accomplishing a stable transformation of *Symbiodinium*.

## Materials and Methods

### *Symbiodinium* and bacterial cell cultures

Dinoflagellate cultures of *Symbiodinium kawagutii* Trench & Blank (from now on referred as *S*. *kawagutii*), *Symbiodinium* sp. Mf11.5b.1 (from now on referred as *S*. Mf11), and *Symbiodinium microadriaticum* Subsp. *microadriaticum* (also known as MAC-CassKB8 and from now on referred to as *S*. KB8) clades F, B and A, respectively, were routinely maintained in our laboratory in ASP-8A medium under photoperiod cycles of 12 h light/dark at 25°C [32]. Light intensity was maintained at 80 μmole quanta m^-2^ sec^-1^. For the success of the transformations, the cultures were required to be axenic. This was achieved by supplementing the ASP-8A medium with 50 μg/ml kanamycin and 5 μg/ml amphotericin B (ASP-8A-M). All cultures were manipulated under sterile conditions at all times until prior to microscopy, or nucleic acid extractions.

*Agrobacterium tumefaciens* (strain GV3101) containing the appropriate plasmids were grown for 16 hr (OD_600_ = 1.5) at 28°C in 5-ml LB medium supplemented with 50 μg/ml kanamycin, 50 μg/ml gentamicin, and 100 μg/ml rifampicin to maintain the selection pressure [33]. These bacterial cultures were used directly for co-incubations with *Symbiodinium* (see below).

### Bacterial strains, plasmid construction, and primers

**Fig 1:**
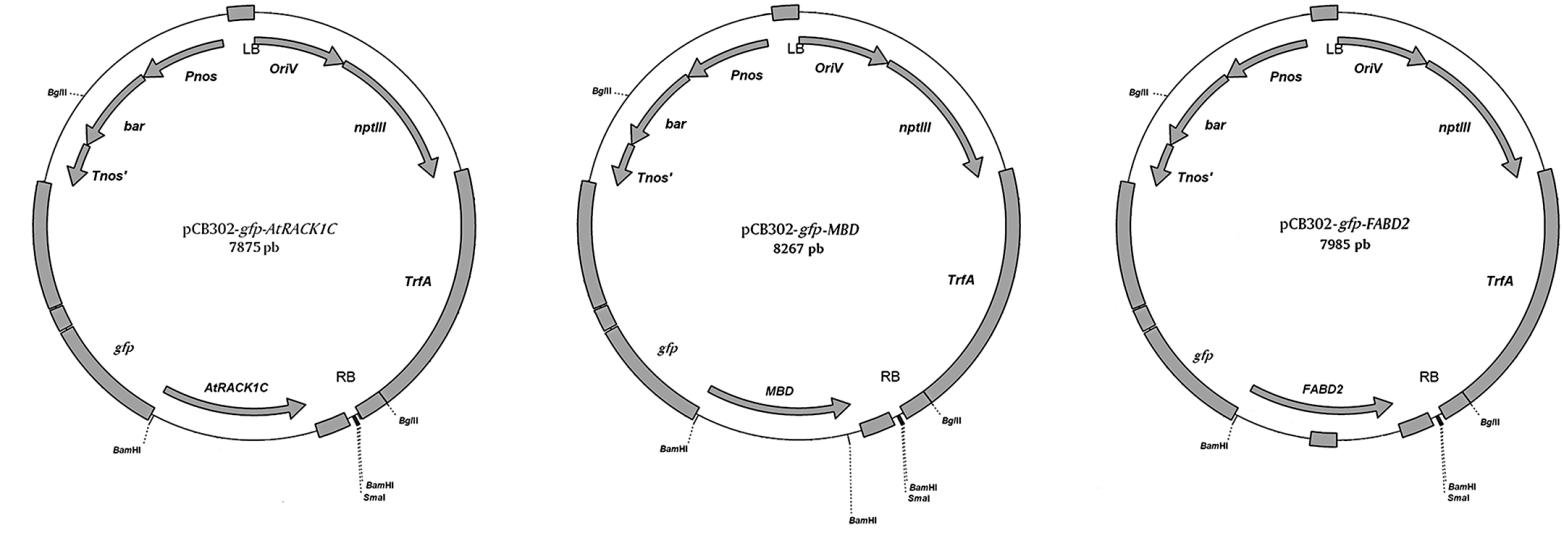
Diagram showing the restriction maps of the different plasmids used in this work. (A) Full-length *gfp*-*AtRACK1C* in the pCB302 plasmid (pCB302-*gfp*-*AtRACK1C*), (B) *gfp*-*MDB* in the pCB302 plasmid (pCB302-*gfp*-*MBD*), and (C) *gfp*-*FABD2* in the pCB302 plasmid (pCB302-*gfp*-*FABD2*).

Cultures of *A*. *tumefaciens* strain GV3101 harboring sequences encoding fusions of *gfp* with a microtubule binding domain (*MBD*); *gfp* with the actin-binding domain 2 of fimbrin (*FABD2*); and *gfp* with *AtRACK1C* from *Arabidopsis thaliana* (pCB302-*gfp*-*MBD*, pCB302-*gfp*-*FABD2*, or pCB302-*gfp*-*AtRACK1C*; Fig 1), were used to transform *Symbiodinium* spp. The cDNA sequence of the *RACK1C* (At3g18130) gene from *A*. *thaliana*, (*AtRACK1C*) was kindly provided as a cloned plasmid in *Escherichia coli* by the Salk Institute (San Diego, CA, USA), and was heat-shock transformed in the *E*. *coli* DH5 α strain. The propagated plasmids were purified and used as template for PCR amplification with the primers: forward, WDF1: 5’-GCGGATCCATGGCCGAGGGACTC-3’; and reverse, 5’-GCACTAGTGTAACGACCAATACCCCA-3’ which amplify the full-length *AtRACK1C* sequence with cutting sites for *Bam*HI at the 5’-end, and for *Spe*I at the 3’-end, in order to create sticky ends. The amplified product was ligated to the plasmid pCATGFPm1 previously cut with *Bam*HI (Fermentas, Glen Burnie, MD) and *Spe*I (Fermentas), in order to join the *gfp* sequence to the N-terminus of the *AtRACK1C* sequence. Thus, the final plasmid construct contained a cauliflower mosaic virus 35S (*CaMV35S*) constitutive promoter with a double enhancer to drive the expression of the GFP-AtRACK1C hybrid protein, and an ampicillin resistance cassette for selection. For the construction of pCB302 with the *gfp-AtRACK1C* sequence insertion, the pCB302 binary plasmid [34] was linearized by cutting at its *Pst*I site and the insert spanning the *gfp-AtRACK1C* coding sequence was excised from pCAT-GFPm1 at its *Sse*8387 sites and ligated with the linearized pCB302 to yield pCB302-*gfp*-*AtRACK1C* (Fig 1) [17]. The correct orientation of the sequence was checked by restriction and gel analysis of the pCB302-*gfp*-*AtRACK1C* construct by agarose electrophoresis. The plasmid was transformed into competent *A*. *tumefaciens* cells for propagation and stock preparation. The pCB302-*gfp*-*FABD2* plasmid was constructed as described in Voigt *et al*., 2005 [35]; and the pCB302-*gfp*-*MBD* plasmid as described in Marc *et al*., 1998 [36].

### *Symbiodinium* spp. co-incubation with *A*. *tumefaciens*

The procedure was carried out by placing 1.3 x 10^7^ *Symbiodinium* cells in 1 ml culture medium in a 2 ml conical bottom plastic tube (Denville Scientific, Inc., Metuchen, NJ) containing a dry volume of 200 μl (approximately 500 mg) acid-washed, sterile glass beads (Cat. No. G8772; 425–600 μm mesh size; Sigma, St. Louis, MO). Then, the suspension was added with 350 μl 20% polyethyleneglycol (PEG-3500; Sigma) and the tube was briefly (90 s) but vigorously shaken (4200 rpm) in a bead beater. After shaking, the cells were transferred to a new sterile 2 ml tube, washed to remove the PEG and added with 150 μl of the *Agrobacterium* culture (OD_600_ = 1.5) harboring the corresponding plasmid, and incubated in fresh culture medium without antibiotics in the dark for one-two days before selection. For the selection of these *A*. *tumefaciens* co-incubated cells, the medium contained 1 mg/ml Basta (Bayer, Research Triangle Park, NC), and to eliminate the *Agrobacterium*, either a 1/10 dilution of a commercial antibiotic solution (final concentrations per ml: 1,000 units penicillin, 1 mg streptomycin, 2.5 μg amphotericin B; Sigma Cat. No. A5955), or a mixture with a final concentration of 50 μg/ml ampicillin and 50 μg/ml kanamycin was used (ASP-8A-sm medium). In addition, 50 μg/ml kanamycin were always present, in order to prevent any other bacterial growth throughout the selection process. The cultures were then maintained in the same solution (final volume 25 ml) in 50 ml sterile tubes under the standard photoperiod conditions and monitored for the appearance of bright-green fluorescence. Cell GFP green fluorescence frequency was based on both, the presence of the Basta-resistant cells, along with the concomitant observation of the characteristic bright-green fluorescence detected by microscopy from triplicate samples from each *Symbiodinium* clade as previously reported [17]. In some cases, tests omitting the PEG and/or the glass beads, or the vigorous shaking, were carried out. In addition, negative controls were carried out by: a) co-incubation with *A*. *tumefaciens* harboring the plasmids plus all the components, except that the brief, vigorous shaking was omitted; b) incubation with all the components plus brief, vigorous shaking, except that *A*. *tumefaciens* was omitted; and c) incubation with all the components, except that *A*. *tumefaciens* and brief, vigorous shaking were omitted.

### Genomic and RT-PCR

Total genomic DNA was extracted by the cetyl trimethylammonium bromide (CTAB) method [37] three weeks after selection. Genomic DNA from either control or *A*. *tumefaciens* co-incubated cultures of *Symbiodinium* were used for PCR analysis with the primer pairs, forward 5’-ACCATGGCCGAGGGACTC-3’ and reverse 5’-CCAACAAGAGAGTTCCTC-3’ to specifically amplify a ~0.6 kbp fragment from the *AtRACK1C* sequence; the same forward and reverse 5’-CTAGTAACGACCAATACCC-3’ to specifically amplify a ~1 kbp fragment from the *AtRACK1C* sequence; or forward 5’-GATGAGTAAAGGAGAAGAAC-3’ and reverse 5’-TATTTGTATAGTTCATCCATGCC-3’ to amplify a ~0.7 kbp fragment from the *gfp* sequence. The PCR amplification was carried out using an Invitrogen Platinum PCR Super Mix kit (Invitrogen-Life Technologies, Carlsbad, CA) in 35 amplification cycles as follows: denaturing at 94°C for 30 s; annealing at 55°C for 45 s, and extension at 72°C for 1 min. For amplification of *gfp* sequences by RT-PCR, RNA from *S*. *kawagutii* cultures which were co-incubated with *A*. *tumefaciens* harboring any of the three vectors, was extracted (11 d post-selection) with the TRI Reagent (Sigma-Aldrich) and remaining DNA was degraded with DNase I (Invitrogen) to assure that only RNA remained. Then the RNA was re-extracted with the TRI Reagent and cDNA was synthesized with M-MLV Reverse Transcriptase (Invitrogen) and used as template. Amplification was carried out with the forward and reverse *gfp* primers for 35 cycles as follows: denaturing at 94°C for 30 s; annealing at 51°C for 30 s, and extension at 72°C for 1.2 min. In addition, 20 μg of the plasmid pCB302-*gfp*-*AtRACK1C* was subjected to the same RNA extraction parameters and used as template to rule out that products arose form bacterial DNA. Parallel positive and negative RT-PCR amplification controls were also performed as follows: a) endogenous *S*. *kawagutii RACK1* amplification with the primers forward 5’-GACATGGCATCCGAGTCCCTCCACTATG-3’ and reverse 5’-CTGCTCGCCCACCCTGTACACGTAGATG-3’ derived from the *S*. KB8 *RACK1* sequence (Villanueva, unpublished; GenBank Accession No. KJ755867.2); and b) amplifications from the three distinct negative controls described above. The PCR products were analyzed by agarose gel electrophoresis on 1% agarose gels run at 80 V and visualized under a UV transilluminator after staining with SYBR Green I (Molecular Probes Inc. Eugene, OR) or GelRed (Biotium, Inc. Hayward, CA). Data were acquired using a Canon PowerShot A640 camera (Canon Inc. Japan). After the analysis, the PCR products were purified from the gel using a S.N.A.P. Gel Purification kit (Invitrogen-Life Technologies), according to the manufacturer’s instructions. The pure PCR products were sequenced at the sequencing facility of the Instituto de Biotecnología, UNAM (Cuernavaca, Morelos, México).

### Microscopy

Cells were observed under phase contrast and epifluorescence under a Zeiss Axioskop 40 microscope (Carl Zeiss, Göttingen, Germany) with 40 X and 63 X objectives under the FITC excitation/emission filter. Data were acquired using a Canon PowerShot A640 camera (Canon Inc., Japan) and the Carl-Zeiss AxioVision software (Carl-Zeiss). Alternatively, CLSM images were acquired using a Zeiss LSM 510 META confocal microscope (Carl Zeiss) equipped with an argon and a helium/neon laser. GFP fluorescence was imaged using excitation with the 488-nm line of the argon laser.

## Results

### Reproducible heterologous gene expression in *Symbiodinium* spp.

**Fig 2:**
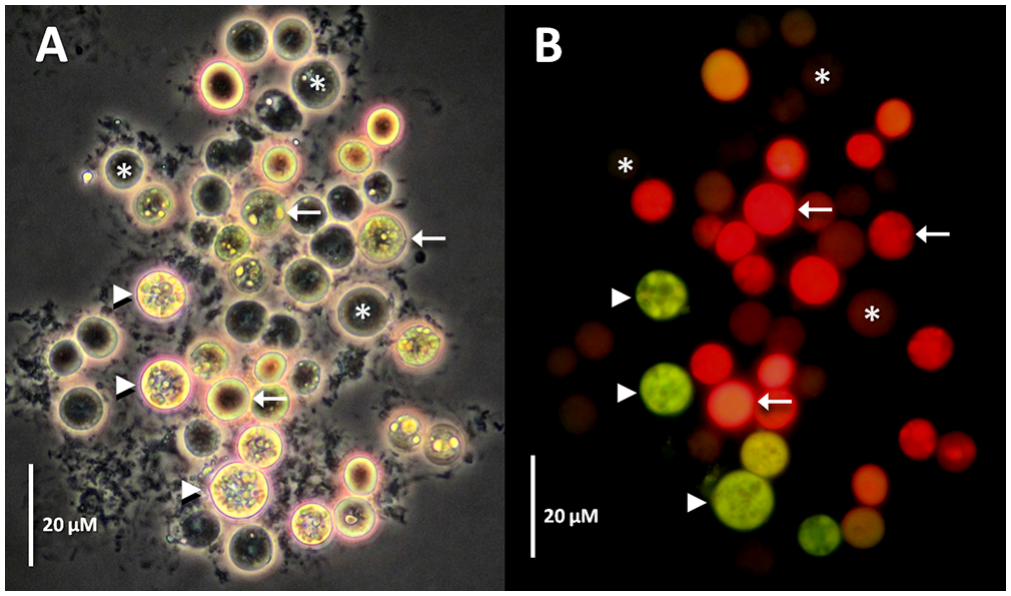
GFP-fusion protein fluorescence from *Symbiodinium kawagutii* cells co-incubated with *Agrobacterium tumefaciens* harboring a *gfp-AtRACK1C* fusion. Distinctive fluorescence pattern between dead (asterisks), dying (arrows) or GFP-fusion protein expressing (arrowheads) *Symbiodinium* cells. The images were obtained under phase contrast (A) or epifluorecence microscopy (B) after 16 d on selection medium. Bars equal 20 μm.

**Fig 3:**
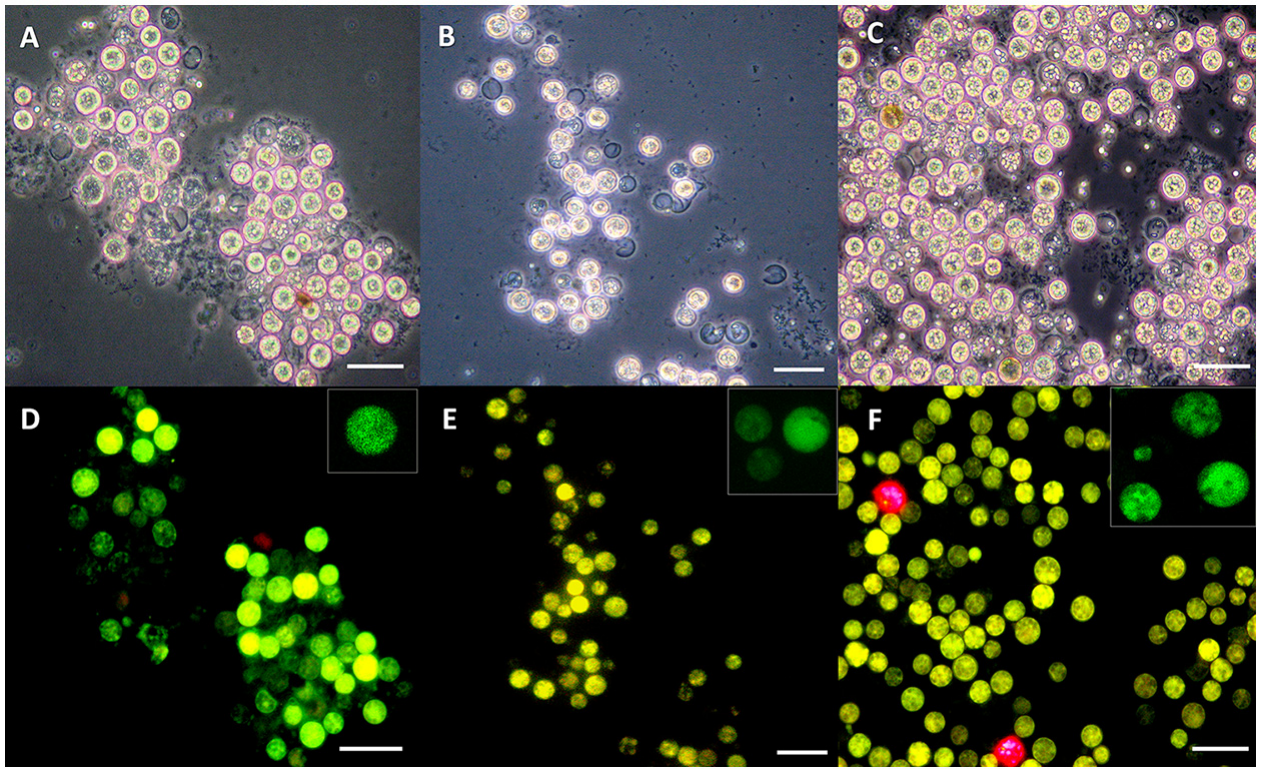
GFP-fusion protein fluorescence from *Symbiodinium* spp. cells co-incubated with *Agrobacterium tumefaciens* harboring a *gfp-AtRACK1C* fusion. Cell cultures of *S*. KB8 (A, D), *S*. Mf11 (B, E), and *S*. *kawagutii* (E, F), after two weeks of selection with Basta. Cells were observed under phase contrast (A, B, and C), and under epifluorescence microscopy (D, E, and F). Bars equal 20 μm. Inset images show cells observed by CLSM.

Since there are several reports documenting transformation methods using *Agrobacterium* to transform genes into a number of cell types including mammalian cells in culture [30] and *Chlamydomonas* [27], we also attempted to use a similar method by co-incubation with *A*. *tumefaciens* (harboring a pCB302-*gfp*-*AtRACK1C* plasmid; Fig 1), in order to obtain heterologous gene expression in *S*. *kawagutii* cells. We monitored them under fluorescence microscopy and detected the characteristic green fluorescence (indicating the expression of GFP) as early as 2 d and throughout selection in ASP-8A-sm medium. Observation under fluorescence microscopy at two weeks of selection allowed us to distinguish between dead (Fig 2B, asterisks), non-expressing (Fig 2B, arrows), and GFP-expressing (Fig 2B, arrowheads) cells, indicating that the green fluorescence was not an artifact of dead cells. Even by phase contrast microscopy, dead/dying cells had obvious differences compared to live cells. Dead/dying cells looked opaque (Fig 2, asterisks), while live cells looked bright with a shiny surface and these bright cells were the ones corresponding to those emitting the green fluorescence (Fig 2, arrowheads). It is important to note, that in some cases, we also had to distinguish between a characteristic bright-green fluorescence from GFP expression in live cells, and an opaque-green autofluorescence from dead cells (see below). Next, we sought to apply our procedure to three different *Symbiodinium* clades including *S*. *kawagutii*. When we applied this method to *S*. KB8, *S*. Mf 11 and *S*. *kawagutii*, we observed green-fluorescent cells in all the clades (*S*. KB8, *S*. Mf11 and *S*. *kawagutii*; Fig 3D, 3E and 3F, respectively, and corresponding insets), indicating the expression of GFP in all of them. We consistently observed that *S*. *kawagutii* cells consistently yielded the highest number of green-fluorescent cells (Fig 3F and Table 1), followed by *S*. KB8, and *S*. MF11, respectively (Fig 3D, 3E and Table 1). In contrast, no green fluorescent cells were detected when no shaking was applied to the *S*. *kawagutii* cells prior to the *A*. *tumefaciens* co-incubation, even at longer selection times (S1B Fig), compared to cells in a parallel co-incubation with shaking (S1D Fig).

**Table 1:** Frequencies of GFP-fusion protein fluorescence detection from *Symbiodinium* KB8, *Symbiodinium* Mf11 and *Symbiodinium kawagutii* after full selection.

*Symbiodinium*	Frequency (per 10^6^ cells)
KB8	570–640
Mf11	421–460
*kawagutii*	790–839

**Fig 4:**
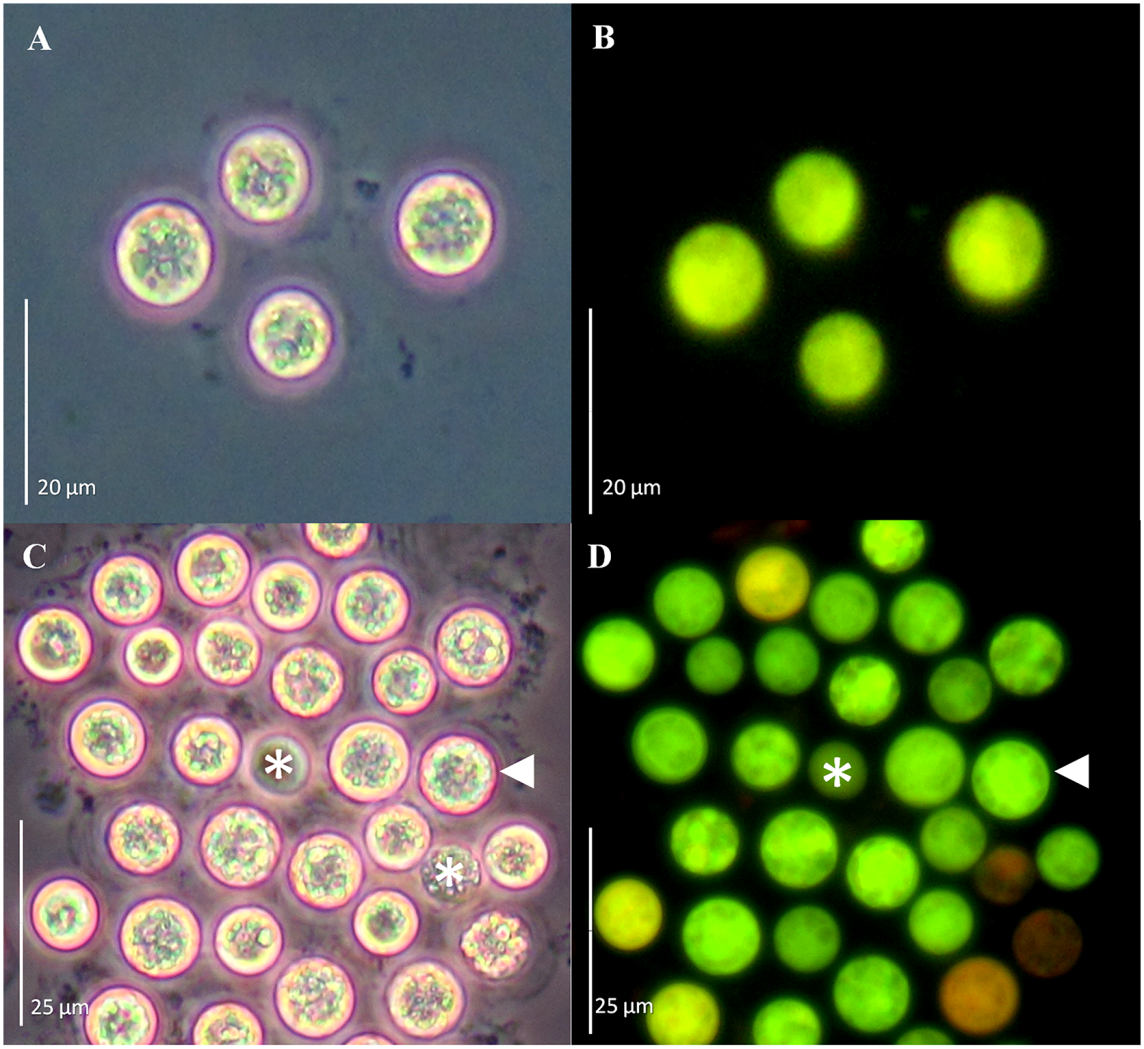
GFP-fusion protein fluorescence from *Symbiodinium* spp. cells co-incubated with *Agrobacterium tumefaciens* harboring a *gfp*-*MBD* fusion. Cell cultures of *S*. Mf11 (A, B), and *S*. *kawagutii* (C, D) after 20 d of selection with Basta. Cells were observed under phase contrast (A and C), and under epifluorescence microscopy (B and D). Bars equal 20 μm for A and B, and 25μm for C and D.

**Fig 5:**
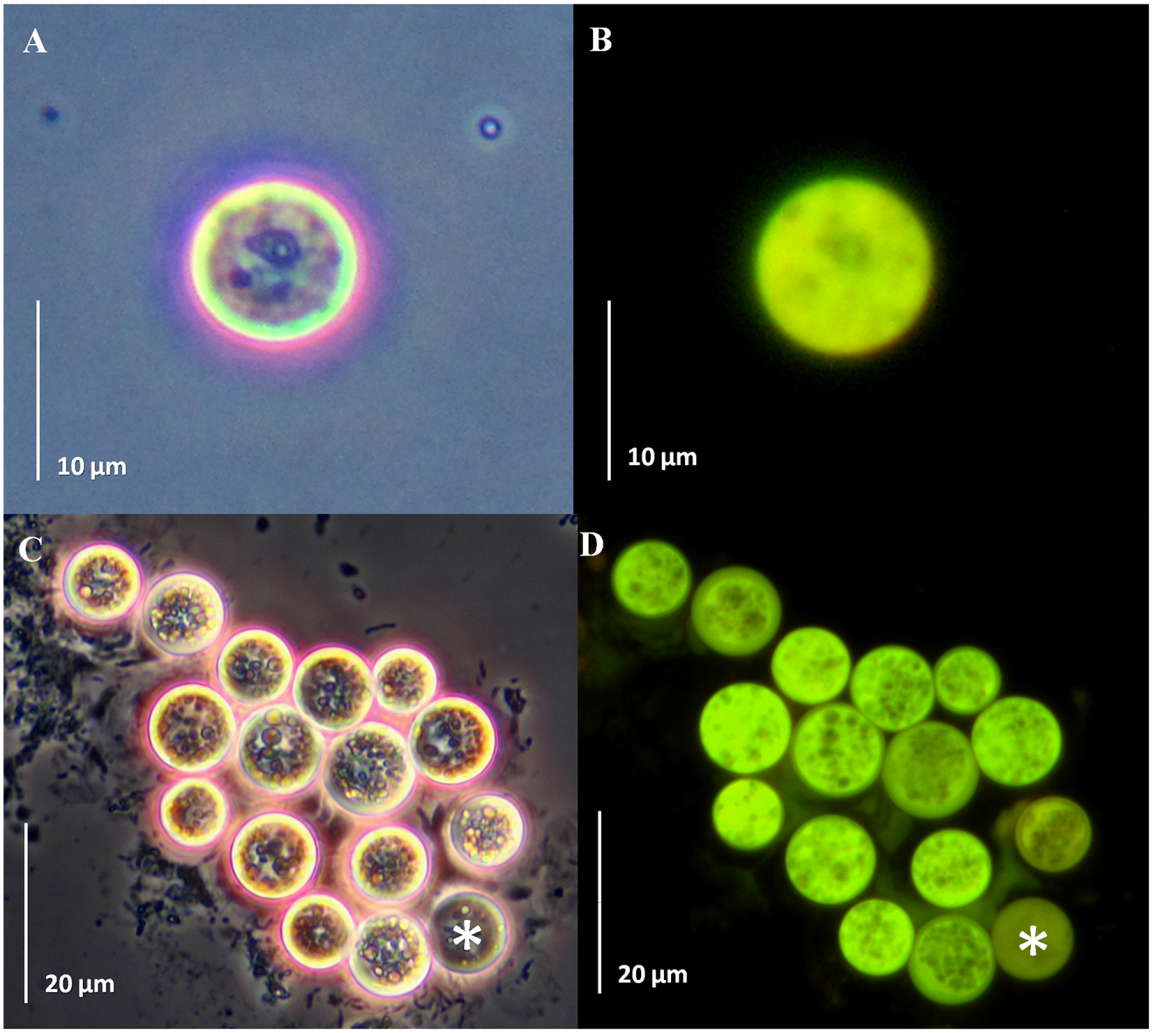
GFP-fusion protein fluorescence from *Symbiodinium* spp. cells co-incubated with *Agrobacterium tumefaciens* harboring a *gfp*-*FABD2* fusion. Cell cultures of *S*. Mf11 (A, B), and *S*. *kawagutii* (C, D) after 23 d of selection with Basta. Cells were observed under phase contrast (A and C) and under epifluorescence microscopy (B and D). Bars equal 10 μm for A and B, and 20 μm for C and D.

The efficiency of this procedure via co-incubation with *A*. *tumefaciens* varied in the range of 421–839 green fluorescent per every 10^6^ cells, within all three clades (Table 1). The cells appeared to enter a quiescent-like phase with no further divisions after co-incubation and selection although they had all the characteristic features of live cells (see Figs 2, 4 and 5). Therefore, this efficiency assessment was based on the presence of the Basta resistant cells, along with the concomitant observation of the characteristic bright-green fluorescence detected by microscopy in triplicate samples from each *Symbiodinium* strain. These data indicated that co-incubation with *A*. *tumefaciens* was successful in aiding the foreign gene expression in different *Symbiodinium* clades in a simple and efficient manner.

To further corroborate the reproducibility of this method, we applied it to *Symbiodinium* spp. cells in culture using two different gene fusion constructs: pCB302-*gfp*-*MBD* and pCB302-*gfp*-*FABD2* (Fig 1). Green-fluorescent *S*. Mf11 and *S*. *kawagutii* cells with the pCB302-*gfp*-*MBD* plasmid (Fig 4B and 4D, respectively) were observed under the epifluorescence microscope after 20 d in selection medium. Similarly, green-fluorescent *S*. Mf11 and *S*. *kawagutii* cells with the pCB302-*gfp*-*FABD2* plasmid construction (Fig 5B and 5D, respectively) were observed after 20 d in selection medium. In both cases, we observed an opaque-green autofluorescence from dead cells (Figs 4C, 4D and 5C, 5D, asterisks) that was clearly different from the bright-green fluorescence from GFP-expressing cells. Furthermore, the opaque autofluorescence appeared diffuse (Fig 4D, asterisk) compared to the expressed GFP fluorescence, which was clearly observed on cellular structures (Fig 4D, arrowhead). These data indicated the reproducibility of the *A*. *tumefaciens* co-incubation method.

### Presence of the heterologous genes in *Symbiodinium* spp. cells

**Fig 6:**
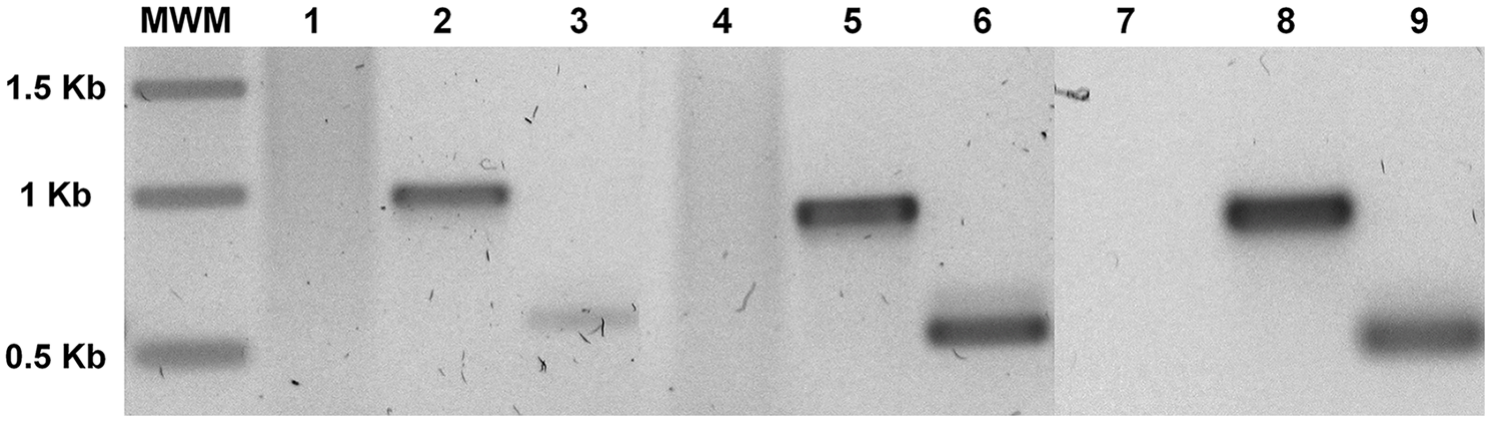
Genomic PCR of cells expressing the *gfp*-*AtRACK1C* construct. Amplification products from genomic DNA extracted from *S*. Mf11 (2, 3), *S*. KB8 (5, 6), and *S*. *kawagutii* (8, 9) co-incubated with *A*. *tumefaciens* harboring the *gfp*-*AtRACK1C* fusion. The gel shows the corresponding ~0.6 kbp and ~1 kbp fragments, respectively, obtained with *AtRACK1C* primers. DNA from control cells did not amplify any PCR product from *S*. Mf11, *S*. KB8, or *S*. *kawagutii* (lanes 1, 4 and 7, respectively). MWM shows the molecular standards. Lanes 1–6 and MWM are from an independent gel from that corresponding to lanes 7–9. The bands on the gel are shown in negative.

**Fig 7:**
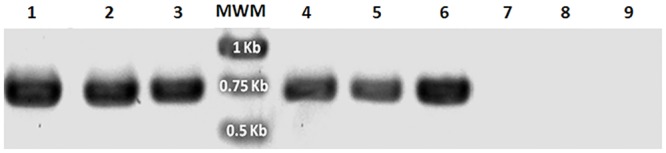
Genomic PCR of cells expressing the *gfp*-*MBD* or *gfp*-*FABD2* constructs. Amplification of a 717 bp fragment using *gfp* specific primers and genomic DNA template from: *S*. *kawagutii* co-incubated with *A*. *tumefaciens* harboring fusions of *gfp-MBD* (lane 1), or *gfp-FABD2* (lane 4); *S*. Mf11 co-incubated with *A*. *tumefaciens* harboring fusions of *gfp-MBD* (lane 2), or *gfp-FABD2* (lane 5); and *S*. KB8 co-incubated with *A*. *tumefaciens* harboring fusions of *gfp-MBD* (lane 3), or *gfp-FABD2* (lane 6). Genomic DNA from control *S*. *kawagutii*, *S*. Mf11, or *S*. KB8 cells did not amplify any PCR product (lanes 7, 8 and 9, respectively). MWM shows the molecular standards. The bands on the gel are shown in negative.

Genomic DNA was isolated from either control, or Basta-resistant *Symbiodinium* cells after *A*. *tumefaciens* co-incubation, and subsequent killing of the bacteria by antibiotic treatment (see Materials and Methods). The genomic DNA was then used as template for PCR amplification using two sets of *AtRACK1C*-specific primers (for *Symbiodinium* cells co-incubated with *A*. *tumefaciens* containing *gfp*-*AtRACK1C*), and one set of *gfp* specific primers (for *Symbiodinium* cells co-incubated with *A*. *tumefaciens* containing *gfp*-*MBD* and *gfp*-*FABD2*). We were able to amplify products of expected size for *AtRACK1C*, with specific primers directed to the *AtRACK1C* sequence and genomic DNA extracted from *S*. *kawagutii*, *S*. Mf11, and *S*. KB8 cells co-incubated with *A*. *tumefaciens* containing *gfp*-*AtRACK1C*. We observed the expected, corresponding bands of ~1 kbp (Fig 6, lanes 2, 5 and 8, respectively) and ~0.6 kbp (Fig 6, lanes 3, 6 and 9, respectively). On the contrary, the same primer sets did not amplify any PCR product when the genomic DNA from control *S*. *kawagutii*, *S*. Mf11, or *S*. KB8 cells was used as template (Fig 6, lanes 1, 4 and 7, respectively). In the case of cells with *gfp*-*MBD*, a PCR fragment was amplified using the specific primer set for the *gfp* sequence. This yielded the expected size product (~0.7 kbp) for *S*. *kawagutii*, *S*. Mf11, or *S*. KB8 (Fig 7, lanes 1, 2 and 3, respectively). Similarly, in the case of cells with *gfp*-*FABD2*, the expected ~0.7 kbp product was also obtained for *S*. *kawagutii*, *S*. Mf11, or *S*. KB8 (Fig 7, lanes 4, 5 and 6, respectively). The same primer set did not amplify any product when genomic DNA from the corresponding control *Symbiodinium* cells was used as template (Fig 7, lanes 7, 8 and 9, respectively). The obtained PCR products were purified and sequenced, and the sequences were identical to those of the *AtRACK1C* or *gfp* sequences inserted in the vector (data no shown). The amplification products were not a result of surviving *Agrobacteria* in the selection medium because inoculated medium on four replicate LB plates without ampicillin revealed growth only immediately after resuspension of the cells in such selection medium (plate sections A in S2 Fig). On the other hand, no growth was detected on the plate after 4 and 12 d of selection (plate sections B and C in S2 Fig, respectively). These results indicated that the *AtRACK1C* and *gfp* sequences remained in the *Symbiodinium* cells and were capable of expression driven by the encoded gene promoters throughout the process.

### Expression of the *gfp* transcript in *S*. *kawagutii* cells

**Fig 8:**
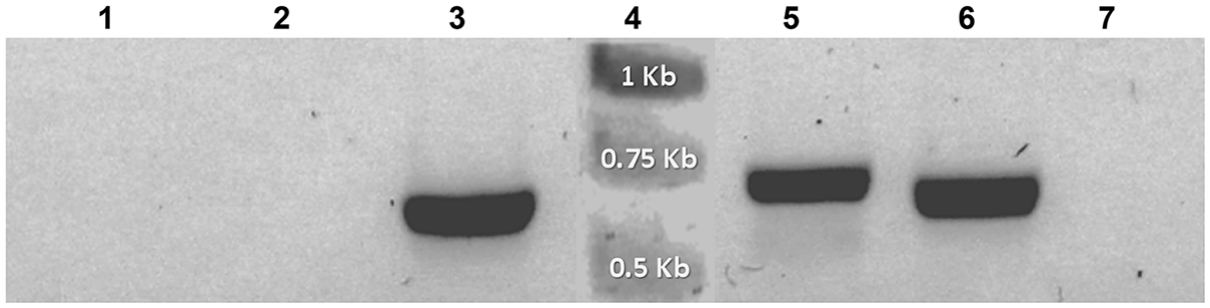
Amplified products from reverse-transcribed cDNA obtained from *Symbiodinium kawagutii* cultures co-incubated with *Agrobacterium tumefaciens* harboring pCB302-*gfp-AtRACK1C*, pCB302-*gfp-MBD*, and pCB302-*gfp-FABD2*. The reverse-transcribed cDNA from *S*. *kawagutii* cultures expressing the *gfp*-fusion constructs was used as template with the *gfp* primers for PCR amplification of the corresponding transcripts (see Materials and Methods). Fragments of ~0.7 kbp corresponding to the *gfp* transcripts were obtained from cDNA of *S*. *kawagutii* co-incubated with *A*. *tumefaciens* harboring: *gfp-AtRACK1C* (lane 3), *gfp-MBD* (lane 5), or *gfp-FABD2* (lane 6). No amplifications were obtained when template cDNA was used from the following negative *S*. *kawagutti* controls: no shaking/no *Agrobacterium* (lane 1); shaking/no *Agrobacterium* (lane 2); or *Agrobacterium*/no shaking (*gfp-AtRACK1C*) (lane 7). The presence of cDNA in preparations from control and *A*. *tumefaciens* co-incubated cells was confirmed by RT-PCR of the endogenous *S*. *kawagutii RACK1* transcript (data not shown). Lane 4 shows the molecular standards. The bands on the gel are shown in negative.

Since the highest number of green fluorescent cells was obtained with *S*. *kawagutii*, we used this clade to further confirm GFP expression. We applied our procedure to *S*. *kawagutii* cells with all three constructs and we extracted their RNA at 11 d of selection in order to obtain the highest amount of material for RT-PCR amplification with the appropriate *gfp* primers. We carried out three negative controls in parallel, from which we also extracted RNA to use as template in the reactions. In all cases, RNA from *A*. *tumefaciens* co-incubated cultures amplified a ~0.7 kbp product (Fig 8, lanes 3, 5 and 6) which, when sequenced, matched the *gfp* sequence (not shown). In contrast, the negative control without shaking but containing *A*. *tumefaciens* harboring the pCB302-*gfp*-*AtRACK1C* plasmid did not produce any amplification (Fig 8, lane 7). Similar results were obtained with negative controls without the bacterium and without shaking (Fig 8, lane 1), or without the bacterium and with shaking (Fig 8, lane 2). These results demonstrated the presence of the *gfp* transcript only in the properly *A*. *tumefaciens* co-incubated *S*. *kawagutii* cells. The presence of cDNA in the preparations obtained from control and *A*. *tumefaciens* co-incubated cells was confirmed by RT-PCR amplification of the endogenous *S*. *kawagutii RACK1* transcript (data not shown), with primers designed from the reported *S*. KB8 *RACK1* sequence (Villanueva, unpublished; GenBank Acc. No. KJ755867.2).

## Discussion

Transformation procedures for cells surrounded by rigid cell walls has proven to be a challenge due to the barriers that prevent the DNA delivery. Plant cells have been the exception since it was discovered that *Agrobacterium*, a gram-negative soil phytopathogen, was capable of delivering foreign genetic material [20]. More recently, *Agrobacterium* was found capable, under laboratory conditions, of genetically transforming non-plant eukaryotic organisms, including cultured human cells [30] demonstrating that *Agrobacterium*-mediated transformation could be applied to other organisms. Thus, it was not so far-fetched to co-incubate *Symbiodinium* cultures with *A*. *tumefaciens* in our attempt to introduce the genes of interest into these dinoflagellates. In order to succeed, it was necessary to obtain access through the rigid cell wall, as well as appropriate resistance and reporter gene expression to monitor the transformation. Since *Symbiodinium* cells are photosynthetic, we used an ammonium glufosinate-based herbicide (Basta), in order to effectively promote cell death in *Symbiodinium* cells not expressing that gene. Glufosinate inhibits the activity of the enzyme glutamine synthetase (GS) resulting in a rapid accumulation of ammonium, inhibition of photosynthesis and cell death [38], whereas expression of the *bar* gene (which encodes the enzyme phosphinothricin N-acetyltransferase), results in the rapid conversion of glufosinate to the non-phytotoxic metabolite N-acetyl- L-glufosinate and confers resistance to the herbicide in cells expressing the enzyme. This was also probably true for the photosynthetic *Symbiodinium* cells since incubation of different clades of wild type *Symbiodinum* cells in the presence of this type of herbicide promoted cell death and, on the contrary, *Symbiodinium* cells that carried the *bar* resistance gene were able to survive after incubation in selection medium with Basta [17]. Indeed, we were able to select the cells expressing the heterologous genes with Basta, which also indicated that appropriate *nos*-driven expression of the *bar* gene within the *Symbiodinium* cells occurred. We also took advantage of the availability of green-fluorescent reporter genes that have been extensively used for plant transformation and *in vivo* monitoring of expression. *Symbiodinium* cells emit red fluorescence and thus, a green-fluorescent reporter would allow the observation of mixed yellow or green fluorescence. Therefore, we used GFP-hybrid proteins to monitor the selection process by fluorescence microscopy. While in selection medium, the cells were monitored by phase contrast and fluorescence microscopy; the dying non-GFP fusion expressing cells were evident by their emission of red fluorescence from chlorophyll and other pigments, even under the GFP filter (see Fig 2). Dead cells without the pigments (due to loss of the chloroplast or pigment degradation) did not emit the characteristic green fluorescence under the GFP filter and their deterioration was obvious by observation under phase contrast (see Fig 2). Nevertheless, some obviously dead cells observed by phase contrast microscopy did show some green autofluorescence under fluorescence using the GFP filter, but it was rather opaque and diffuse and did not localize to cellular structures as in the case of the GFP fusion-expressing cells (Fig 4C and 4D, asterisk and arrowhead, respectively). On the other hand, cells expressing GFP fusions were detected as yellow or green fluorescent (Figs 2–5). It should be noted that the overexpression of GFP-fused proteins produced fluorescent green-looking cells under the epifluorescence microscope at the end of the selection; this was unusual since the combination of the fluorescent red chlorophyll and fluorescent green GFP should have resulted in fluorescent yellow-looking cells. In addition, we observed both, intermediate fluorescence and intermediate pigmentation in the cells during the selection process. Although we have no data regarding the reason for this observation, one possibility is that a strong promoter such as *CaMV35S*, driving the overexpression of the GFP-fused proteins may affect the pathways leading to the synthesis of chlorophyll and/or similar photosynthetic pigments. The actual effect of overexpression of the heterologous genes on the *Symbiodinium* cells, and its possible involvement in photosynthetic pigment metabolism merits further research. Perhaps the use of endogenous constitutive or inducible promoters could alleviate this problem and we are actively searching for such promoters.

We were able to obtain reproducible and observable GFP fusion protein expression with different gene constructs (*gfp*-*AtRACK1C*, *gfp*-*MBD* and *gfp*-*FABD2*), in three different clades of *Symbiodinium* cells (*S*. KB8, *S*. Mf 11, and *S*. *kawagutii*), by co-incubating them with *A*. *tumefaciens* containing the plasmids with the GFP fusion proteins that we wanted to express. Only two reports of *Symbiodinium* transformation exist, one using SiC whiskers [16], and our recently reported glass bead-mediated transient transformation method [17]. However, the SiC whiskers procedure yielded a lower efficiency of transformation (5–24 transformants per 10^7^ cells) compared to either of our methods ([17] and Table 1 in this work). In fact, SiC whiskers have been reported to yield a low transformation efficiency [39], and they represent a health hazard [40]. Although we cannot assure that we have achieved stable transformation, the present *Agrobacterium*-aided DNA uptake procedure represents a significant advance over these previous methods. The relatively high efficiency of this procedure is consistent with the report of Kumara et al. [27] for *Chlamydomonas reinhardtii* transformation, where the transformation frequency using *Agrobacterium* was 50-fold higher than that of their glass bead transformation method. In this work, the highest number of cells expressing GFP fluorescence per every 10^6^ cells was obtained with *S*. *kawagutii*, probably due to their higher stability observed throughout the harsh process of bead beating and selection, compared to the other phylotypes. Therefore, this species has the potential for providing a good model to carry out stable transformation and functional genomics studies.

As reported previously for the expression of a GUS reporter gene [16], we also found that the *CaMV35S* and *nos* promoters were efficient to drive the expression of the gene construct harboring the *gfp*-fused sequences and thus, can be used to constitutively express any desired sequence in *Symbiodinium* cells.

We were able to specifically amplify products with identical sequence to those of the fused genes using genomic DNA from the three different GFP-fusion protein expressing clades as template, and distinct specific sets of primers (Figs 6 and 7), indicating the presence of the introduced heterologous gene sequences in the *Symbiodinium* cells. These PCR products were not the result of surviving bacteria in the selection medium since no bacterial growth was detected in LB plates at a time as early as 4 d of selection (S2 Fig).

Since the cells appeared to enter a quiescent-like phase after transformation although still alive, we could not obtain enough material for assessment of expression using protein extracts. To maximize the amount of material and increase the chances of successfully detecting expression, we used the higher heterologous gene expression frequency found in *S*. *kawagutii* cultures and RT-PCR from their RNA obtained at a relatively early selection stage. Thus, we were able to obtain DNA-free RNA from *S*. *kawagutii* cells expressing GFP-fusion proteins after 11 d of selection, and use the corresponding cDNA as template to detect *gfp* transcripts. Indeed, this approach allowed us to identify specific RT-PCR products of the expected size (Fig 8) corresponding to the *gfp* sequence (not shown), indicating the presence of the *gfp* transcript within the *A*. *tumefaciens* co-incubated *S*. *kawagutii* cells. The specificity of the amplified products obtained from the RT-PCR reactions was corroborated with three distinct negative controls (Fig 8). The amplifications were not due to the absence of cDNA template in the control cell preparations since both cDNA from control and from heterologous gene-containing *A*. *tumefaciens* co-incubated cells were able to RT-PCR amplify endogenous *S*. *kawagutii RACK1* (data not shown). Furthermore, the amplified products were not due to the presence of plasmid DNA from the bacteria because no amplifications were obtained with: a) template from negative *A*. *tumefaciens* co-incubation controls (without shaking) containing the bacteria (Fig 8, lane 7); and b) template from *A*. *tumefaciens* plasmid DNA digested with DNase I under the same conditions used for RNA and using the *gfp* primers, thus demonstrating the complete elimination of bacterial DNA by the DNase I treatment.

In spite of all the above results, the present evidence cannot fully demonstrate *Agrobacterium*-mediated genomic integration. We can only provide evidence that co-incubation with the bacteria after brief, vigorous shaking produces a higher frequency of GFP-expressing green fluorescent cells compared to that of transient transformations with naked DNA [17], and compared to that without shaking, in which no DNA uptake occurs. This suggests that abrasion of the wall significantly enhances the entry of the heterologous DNA in the presence of *A*. *tumefaciens*. Whether such DNA is maintained as a stable plasmid capable of expression, or is integrated in the *Symbiodinium* genome, remains to be determined.

Finally, it is important to note several facts that made the procedure reproducible. First, many tests were carried out in which either the glass beads, or the PEG, or both were omitted in the protocol, as well as tests without shaking in the presence of *A*. *tumefaciens* harboring the vectors containing the *gfp*-fusion sequences. In all cases, similar results of a marginal or null DNA uptake, evidenced by no GFP expression, were obtained (S1B Fig). The presence of all compounds, as well as the accessibility to the cell through cell wall abrasion by shaking with the glass beads appear to be important. This indicates that the delivery of the genes by the *Agrobacteria* requires the opening and/or loosening of the *Symbiodinium* cell wall. Next, even though we do not know whether *vir* gene-mediated entry of plasmid occurs, acetosyringone treatment [41] did not increase the number of GFP-fusion protein expressing cells compared to an untreated control (data not shown). Third, the cultures had to be rendered axenic by the use of antibiotics in order to obtain a successful and contaminant-free selection. This was possible because it is known that *Symbiodinium* cultures are resistant to several antibiotics [16, 42]. Fourth and last, in usual transformed plant selection with Basta, a concentration of 20 mg/L herbicide is commonly used [43]. For the selection of the *bar* gene-expressing *Symbiodinium* cells, we raised the concentration of Basta to 1 g/L in order to achieve a good selection [17].

New genetic transformation methods for different organisms are important, not only to introduce genes of interest for industrial and biotechnological applications, but also to carry out functional genomics research. In recent years, the number of reports on different eukaryotic organisms that are susceptible to uptake of heterologous DNA aided by *Agrobacterium* has increased [44], and now, we have added *Symbiodinium* to this growing list. Our present work further confirms that *Agrobacterium*-aided heterologous DNA uptake is a viable method for introducing genes of interest to a wide range of cellular species. Functional genomics approaches have not yet been applied to *Symbiodinium* precisely due to the lack of a reproducible and reliable transformation method for these cells, and although this reported procedure still presents the severe limitation that cells do not futher divide after the DNA uptake, it represents an important first step towards achieving stable *Symbiodinium* transformations.

## Supporting Information

S1 Fig Heterologous DNA uptake procedure with or without brief vigorous shaking in the presence of glass beads. Micrograph showing *S*. *kawagutii* cells when the complete DNA delivery protocol was applied (C and D) or when brief, vigorous shaking in the presence of glass bead was omitted (A and B). Cells were observed under phase contrast (A and C), and under epifluorescence microscopy (B and D) after 19 d in selection medium. Bars equal 40 μm for A and B, and 35 μm for C and D.
(TIF)

S2 Fig Assay of *Agrobacterium tumefaciens* growth on LB plates from sequential selection times after application of the heterologous DNA uptake procedure. An aliquot of the supernatant from the *A*. *tumefaciens-S*. *kawagutii* (up) and *A*. *tumefaciens-S*. Mf11 (down) co-incubated cells was streaked on LB plates with kanamycin and gentamycin after they were resuspended in selection medium plus ampicillin to kill the bacteria (A). Further inoculations were performed after 4 (B), and 12 (C) d after selection. In all cases, bacterial growth was only observed at the initial inoculation but the bacteria did not survive after 4 d or further in selection medium with ampicillin.
(TIF)
