# Supplementary figures and images for: Heterologous DNA Uptake in Cultured Symbiodinium spp. Aided by Agrobacterium tumefaciens
Source: PLoS One. 2015 Jul 13;10(7):e0132693. doi: 10.1371/journal.pone.0132693 (PMC4500500; doi:10.1371/journal.pone.0132693)

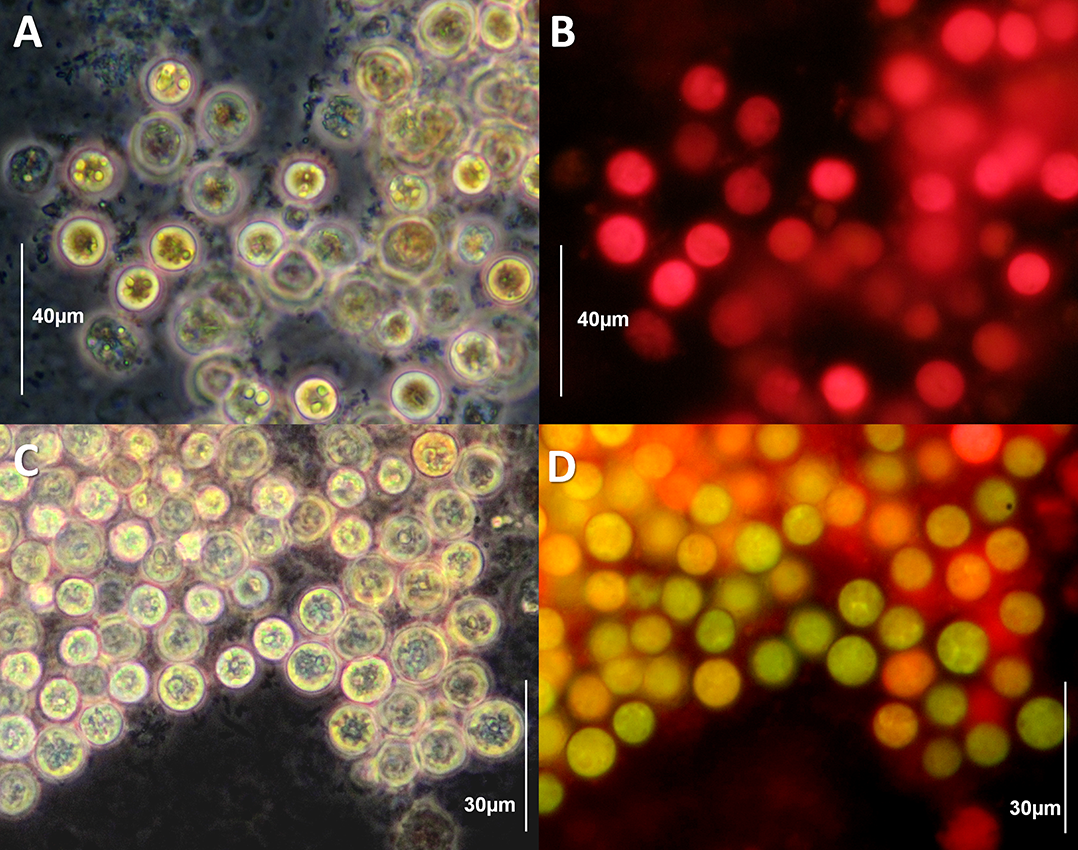

Supplement: S1 Fig — Micrograph showing S. kawagutii cells when the complete DNA delivery protocol was applied (C and D) or when brief, vigorous shaking in the presence of glass bead was omitted (A and B). Cells were observed under phase contrast (A and C), and under epifluorescence microscopy (B and D) after 19 d in selection medium. Bars equal 40 μm for A and B, and 35 μm for C and D. (TIF) [file pone.0132693.s001.tif]

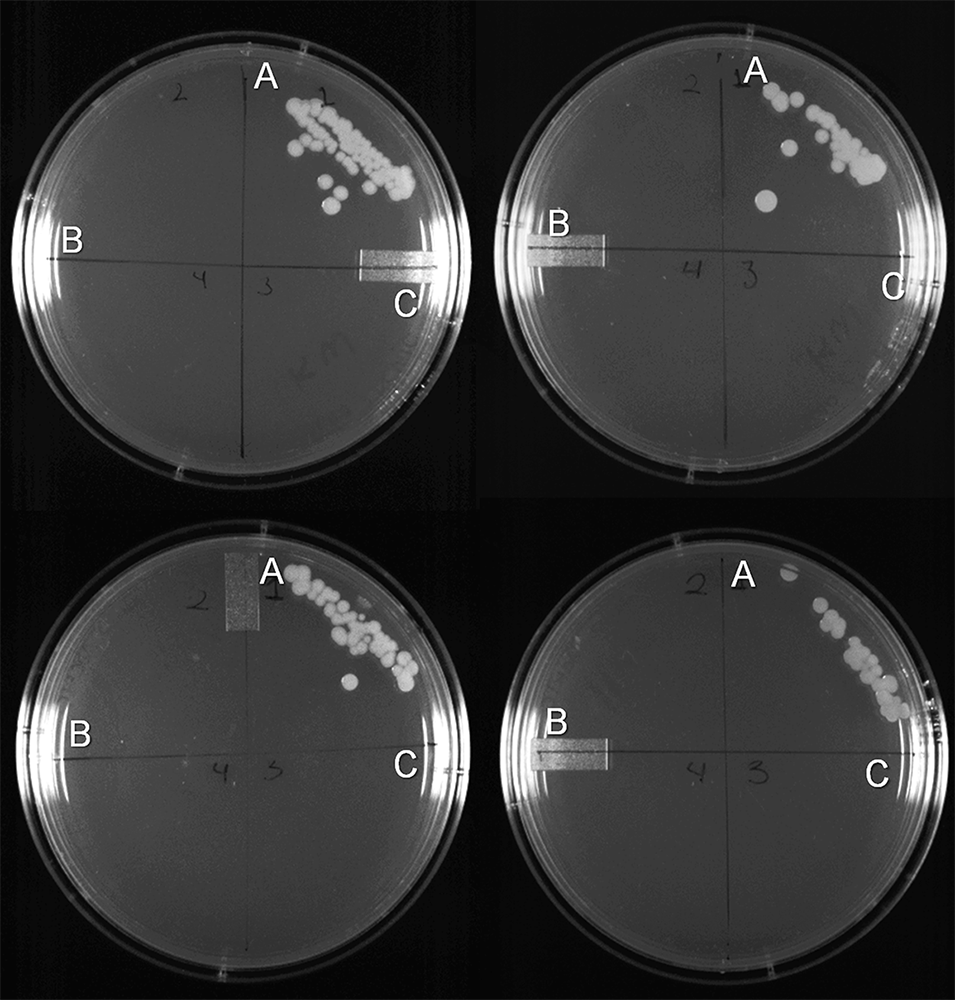

Supplement: S2 Fig — An aliquot of the supernatant from the A. tumefaciens-S. kawagutii (up) and A. tumefaciens-S. Mf11 (down) co-incubated cells was streaked on LB plates with kanamycin and gentamycin after they were resuspended in selection medium plus ampicillin to kill the bacteria (A). Further inoculations were performed after 4 (B), and 12 (C) d after selection. In all cases, bacterial growth was only observed at the initial inoculation but the bacteria did not survive after 4 d or further in selection medium with ampicillin. (TIF) [file pone.0132693.s002.tif]
